# Targeting HMGB1 in endothelial cells reverses heme-induced SIRS after radiofrequency ablation of hepatic hemangioma

**DOI:** 10.3389/fimmu.2025.1680433

**Published:** 2025-11-06

**Authors:** Changyu Yao, Ying Zhou, Liuyang Yu, Li Xu, Shilun Wu, Ruize Gao, Yirui Hou, Wenbing Sun, Jun Gao, Shaohong Wang

**Affiliations:** 1 Department of Hepatobiliary Surgery, Beijing Chaoyang Hospital, Capital Medical University, Beijing, China; 2 Department of Pathology, Peking University People’s Hospital, Peking University, Beijing, China; 3 Department of Anesthesiology, Peking University People’s Hospital, Peking University, Beijing, China

**Keywords:** endothelial cells, HMGB1, heme, radiofrequency ablation, SIRS

## Abstract

**Background:**

Although radiofrequency ablation (RFA) is a safe and effective treatment for hepatic hemangiomas, post-RFA systemic inflammatory response syndrome (SIRS) frequently occurs. The role of high-mobility group box 1 (HMGB1) in endothelial cell pyroptosis and SIRS induction following RFA in hepatic hemangiomas remains unexplored.

**Methods:**

*In vitro*, the levels of interleukin (IL)-1β, IL-18, and pyroptosis markers, such as GSDMD-N and Casp1 p20, were measured in human umbilical vein endothelial cells (HUVECs) after heme administration. *In vivo*, an orthotopic liver hemangioma mouse model was established, and RFA was performed to evaluate the levels of IL-1β and IL-18, wet-to-dry lung ratio, and inflammation score. In addition, hemopexin and glycyrrhizin were used to investigate the impact of HMGB1 on heme-induced SIRS post-RFA in hepatic hemangioma mice.

**Results:**

Heme induced elevated levels of IL-1β and IL-18, endothelial cell death *in vitro*, and increased wet-to-dry lung ratio and inflammation score *in vivo*. These effects were rescued with the administration of heme-binding protein hemopexin, indicating the role of heme in inducing SIRS and pyroptosis post-RFA of hepatic hemangioma. HMGB1 participates in heme-induced SIRS in mice by regulating HMGB1/nod-like receptor family pyrin domain-containing 3 (NLRP3) pathway through reactive oxygen species (ROS). Treatment with hemopexin or the HMGB1 inhibitor glycyrrhizin reversed heme-induced SIRS after RFA of hepatic hemangioma in mice.

**Conclusions:**

Collectively, we demonstrated that heme induces SIRS through the ROS/HMGB1/NLRP3 pathway-regulated endothelial cell pyroptosis in mice, and hemopexin, a heme scavenger, and glycyrrhizin, a HMGB1 inhibitor, may be the potential strategies for further study for SIRS following the RFA of hepatic hemangioma for the first time.

## 1 Introduction

Hepatic hemangiomas are the most common benign liver tumors (1). In recent years, radiofrequency ablation (RFA) has been used to treat hepatic hemangiomas, demonstrating a significant curative effect with minimal invasiveness and safety advantages comparable to surgical resection (2). However, hemolysis follows RFA in nearly all cases, attributed to the abundant blood supply of hepatic hemangiomas, and represents a significant drawback (3, 4). The released hemoglobin is broken down into heme, which poses a risk of systemic inflammatory response syndrome (SIRS), manifested by increased temperature, respiration, heart rate, and white blood cell count in the short term (5). Our previous study showed that heme plays a key role in SIRS after RFA in hepatic hemangiomas (6). However, the underlying pathophysiology of heme-induced SIRS after hepatic hemangioma ablation has not been fully elucidated.

Pyroptosis is a form of programmed cell death characterized by gasdermin D (GSDMD) cleavage, rapid plasma membrane rupture, and the release of inflammatory cytokines, such as interleukin (IL)-1b, IL-18, and other intracellular contents. Activation of pyroptosis can induce septic shock, multiple organ dysfunction syndrome, or increase the risk of secondary infections (7, 8). High-mobility group box 1 (HMGB1) is a protein primarily found in the cell nucleus that serves as a damage-associated molecular pattern (DAMP) and regulates innate immune responses in both the intracellular and extracellular settings (9). Moreover, elevated circulating HMGB1 level can significantly contribute to systemic inflammation and multiple organ injury (10). Recent studies have shown that HMGB1 activates the NOD-like receptor family pyrin domain-containing 3 (NLRP3) inflammasome, leading to hepatocyte pyroptosis and the subsequent progression of inflammation and tissue injury (11). However, whether HMGB1 participates in heme-induced endothelial cell pyroptosis and SIRS after RFA in hepatic hemangiomas remains unexplored. While several HMGB1 inhibitors are available, glycyrrhizin was selected not only for its ability to reduce HMGB1 expression, but also for its capacity to regulate key inflammatory pathways. Notably, glycyrrhizin has been shown to decrease pro-inflammatory cytokine release, suggesting a potential link to pyroptosis (12). However, its protective role in heme-induced SIRS after RFA of hepatic hemangiomas has not been reported.

The present study aimed to investigate whether heme-induced SIRS occurs through an HMGB1-dependent pathway in endothelial cells following RFA for hepatic hemangioma. Our findings revealed that heme-induced HMGB1 upregulation leads to endothelial cell pyroptosis, resulting in SIRS following RFA of hepatic hemangioma. In addition, our study indicates that hemopexin, a heme scavenger, and glycyrrhizin, a HMGB1 inhibitor, could alleviate SIRS after RFA of hepatic hemangioma. Overall, this study elucidates the mechanisms and potential targets of SIRS in hepatic hemangiomas following RFA.

## 2 Materials and methods

### 2.1 Cell culture

Human umbilical vein endothelial cells (HUVECs) were purchased from the Institute of Biochemistry and Cell Biology, Chinese Academy of Sciences. HUVECs were cultured in complete ECM medium supplemented with 1% ECGS, 100 U/mL penicillin/streptomycin, 5% FBS, and 100 μg/mL streptomycin at 37°C with 5% CO2. For the heme stimulation experiment, HUVECs were treated with 40 μM heme for 24 hours. Cell lysates and culture medium were collected for further analysis.

### 2.2 Transfection experiments

Lentivirus-mediated shRNA HMGB1 vector and NLRP3-overexpression vector, were obtained from GeneChem (Shanghai, China). HUVECs were seeded in complete ECM medium and transfected with lentivirus when the cells reached 50%–60% confluency, following the manufacturer’s instructions. An empty vector was used as a negative control. The medium was changed to fresh ECM after 12 hours. Green fluorescent protein expression was observed using fluorescence microscopy three days post-transfection. Transfected HUVECs were then collected for further analysis. The knockdown efficiency of the target genes was assessed by Western blotting. The target sequences used for knockdown were as follows:

sh-HMGB1:5′-GATGCAGCTTATACGAAATAA-3′.

### 2.3 ELISA

The concentrations of IL-1β (Abcam, ab197742) and IL-18 (R&D, DY7625) were measured in mouse plasma samples. Similarly, The levels of IL-1β (Abcam, ab100562), IL-18 (NOVUS, KA0561) and HMGB1 (Arigo Biolaboratories, ARG81351) of HUVECs conditioned medium were determined using ELISA kits following the guidelines provided by the manufacturers.

### 2.4 Measurements of serum heme and reactive oxygen species

Serum heme levels were measured using an Abcam Hemin assay kit (ab65332) according to the manufacturer’s protocols. The amounts of free radicals in serum samples were measured by the OxiSelect *In Vitro* ROS/RNS Assay Kit (Cell Biolabs, STA-347) which uses stabilized dichloro-dihydro-ffuorescein (DCFH) instead of DCFH-diacetate. According to the manufacturer’s protocol, stabilized DCFH is oxidized by ROS. This reaction is catalyzed by a proprietary catalyst included in the assay kit to enhance the oxidation rate. Serum samples were diluted 1:100 in PBS and 50 μL of the dilution was assayed according to the manufacturer’s protocol. The ratio of the fluorescence intensities of oxidized DCFH to DCF is proportional to the concentration of ROS. Fluorescence was assayed at 492 nm excitation/535 nm emissions by Wallac Victor fluorescence plate reader (Perkin Elmer, Massachusetts, USA).

### 2.5 Western blot analysis

The HUVECs were disrupted in radioimmunoprecipitation assay lysis buffer supplemented with protease and phosphatase inhibitors (Solarbio). The cellular protein content was quantified using a bicinchoninic acid (BCA) protein quantitation kit. Protein samples were separated using sodium dodecyl sulfate–polyacrylamide gel electrophoresis and transferred onto a nitrocellulose membrane. The membrane was then blocked using 5% non-fat milk for 1 hour before incubation with the primary antibody at 4°C overnight. Primary antibodies used in this assay were as follows: GSDMD (Abcam, ab210070), GSDMD-N (Abcam, ab215203), Caspase1 (Abcam, ab207802), Caspase1 p20 (CST, 4199T), NLRP3 (Abcam, ab263899), HMGB1 (Abcam, ab18256), ASC (CST, 67824). Protein signals were then achieved using SuperSignal West Pico substrate (Thermo Scientific, Rockford, IL, USA). Subsequently, the quantification of protein signals was carried out using ImageJ.

### 2.6 Immunofluorescence

Lung fixed in 4% PFA for 24 h were washed in PBS and immersed in 30% sucrose for cryoprotection until tissue sank, then frozen in OCT. Lungs were sectioned using Leica cryostat into thin sections. Selected lung sections were washed in PBS, mounted on glass slides, blocked and permeabilized with 10% normal goat serum, 1% Triton in PBS for 1 h at room temperature. Lungs were incubated in the following primary antibodies overnight at 4°C: Anti-NLRP3 (ab270449) and Anti-CD31 (ab7388). Slides were then washed 3 times with PBS and incubated with secondary antibodies for another 2 h at room temperature. DAPI was used to stain cell nuclei. Sections were observed using an inverted fluorescence microscope equipped with an Olympus Qcolor 3 digital camera.

### 2.7 Detection of cell death

After different stimulations of HUVECs, PI (2 μg/mL) and Hoechst (5 μg/mL) were introduced into the cell culture medium and allowed to incubate for 10 minutes at room temperature. Subsequently, cell images were captured randomly using a fluorescent cell imager. To assess cell death, the release of lactate dehydrogenase (LDH) was measured utilizing an LDH cytotoxicity assay kit following the manufacturer’s guidelines. Briefly, a total of 120 μL of cell supernatant and 60 μL of the prepared LDH reagent were combined in a 96-well plate, then incubated on a horizontal shaker in the dark for 30 minutes. Following incubation, the absorbance of the samples was read at 490 nm using a NOVOstar Microplate Reader.

### 2.8 Detection of intracellular ROS

Intracellular levels of reactive oxygen species (ROS) in HUVECs were assessed using the DCFH-DA fluorescent probe. Heme-incubated cells were then treated with DCFH-DA (10 μM) for 30 min in the absence of light. Subsequently, cell images were captured randomly using a fluorescent cell imager.

### 2.9 Mice experiment

Pathogen-free mice that were 8–12 weeks old were utilized. The mice were randomly divided into the following three groups: PBS group, heme group, heme + hemopexin group. Heme (Sigma-Aldrich, St. Louis, MO, USA) was injected intraperitoneally at a dose of 20 µM/kg body weight. In the heme + hemopexin group, hemopexin was injected 5 mg/kg for 3 days before heme injection. Serum and lungs were collected from mice 24 h after treatment.

### 2.10 Murine hepatic hemangioma model and RFA *in vivo*

30 μL suspension, consisting of H5V endothelial cells (5 × 10^6^) and Matrigel, was injected into the subcapsular region of the liver parenchyma in the median lobe of mice. The tumor volume was monitored at specified time points until it reached a size of around 1 cm, following which radiofrequency ablation (RFA) was conducted. Mice were randomly divided into the following four groups: sham group, RFA group, RFA + glycyrrhizin group (50 mg/kg/d), and RFA + hemopexin group (5 mg/kg/d). The radiofrequency current generator (Covidien, Mansfield, MA, USA) was used to generate radiofrequency energy. A 17-gauge cool-tip electrode of 15 cm length with 0.7 cm exposed tip (Covidien, Mansfield, MA, USA) was utilized to deliver the radiofrequency energy. Each ablation cycle lasted for 5 seconds with a power output of 5 W and 10 cycles were performed on each mouse. Mice were pretreated with hemopexin or glycyrrhizin for 3 days respectively before RFA of hepatic hemangioma. Serum and lungs were collected from mice 24 h after RFA. 1 mL of the glycyrrhizin solution was composed of 100 μL of DMSO stock solution (25 mg/mL) and 900 μL of 20% Sulfobutylether-β-Cyclodextrin (SBE-β-CD) saline solution, and 1 mL of the vehicle solution was composed 100 μL of DMSO and 900 μL of the 20% SBE-β-CD saline solution.

### 2.11 Inflammation score

The inflammation score was determined following the previously established method (13). Symptoms, such as periorbital exudates, tremors, lethargy, respiratory distress, diarrhea, and piloerection, were monitored after heme injection or RFA of hepatic hemangioma. Each symptom was assessed and scored as either 1 or 2 based on its severity or presence.

### 2.12 Wet-to-dry lung ratio

The isolated lungs were weighed (wet weight). Then, the lungs were dried in an oven at 60°C for 48 h and measuring their weight again after drying (dry weight). The wet weight and dry weight measurements were then used to calculate the wet-to-dry lung ratio.

### 2.13 Lung histology scoring

Tissues from the right lung of mice were made into paraffin-embedded sections, which were then stained with hematoxylin for 5 min and counterstained with eosin for 3 min. The sections were observed under microscope with lung tissue evaluated and graded blindly by applying an arbitrary grading scale ranging from 0 to 8. Briefly, the following pulmonary parameters were assessed, including alveolar neutrophil infiltration, alveolar hemorrhage, alveolar edema, alveolar epithelial necrosis, as well as interstitial and perivascular cells infiltration (14).

### 2.14 Statistical analysis

The statistical data were presented as the mean ± SEM. A two-tailed Student’s *t*-test or one-way ANOVA variance analysis was conducted to calculate statistically significant differences. Statistical significance was defined as *P* < 0.05. GraphPad Prism version 8 was used to conduct all statistical analyses in this study.

## 3 Results

### 3.1 Hemopexin reverses heme-induced pyroptosis of endothelial cells

**Figure 1:**
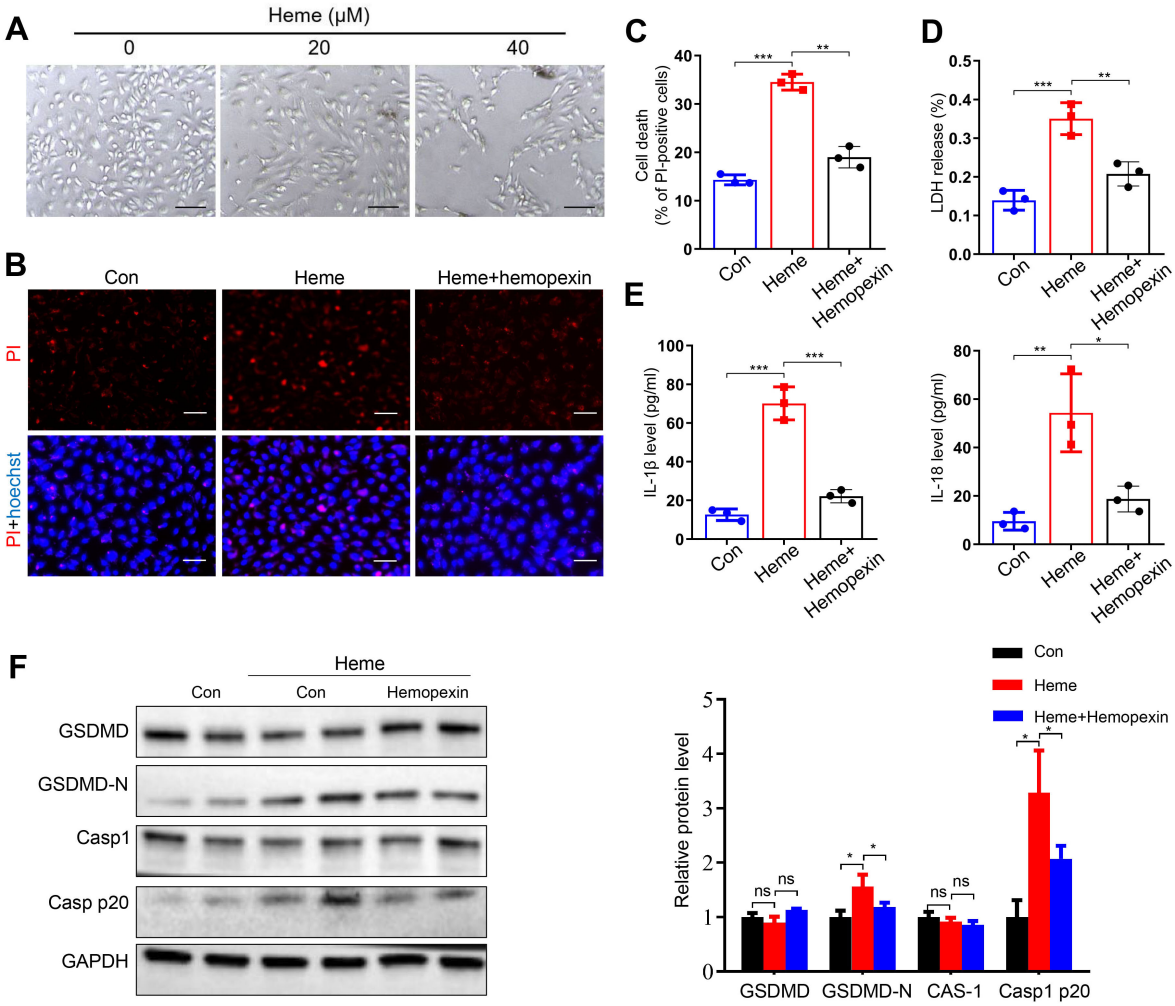
Hemopexin inhibits heme-induced pyroptosis of HUVECs. **(A)** The morphological changes of HUVECs were observed at 24 h after heme treatment. Scale bar = 100 µm. **(B)** The HUVECs were stained with PI and Hoechst for 20 min to observe cell death. Scale bar = 100 µm. **(C)** Cell death of HUVECs with treatment of heme and hemopexin was assayed (n=3, by one-way ANOVA). **(D)** The LDH level in supernatant of HUVECs with treatment of heme and hemopexin was detected (n=3, by one-way ANOVA). **(E)** Levels of IL-1β and IL-18 in supernatant of HUVECs with treatment of heme and hemopexin were assayed by ELISA (n=3, by one-way ANOVA). **(F)** Protein levels of GSDMD, GSDMD-N, Casp1, and Casp1 p20 in HUVECs with treatment of heme and hemopexin were detected by Western blot assay (n=3, by one-way ANOVA). The data are expressed as mean ± SEM. ns: no significance, *P < 0.05, **P < 0.01, ***P < 0.001.

Heme-induced morphological changes in HUVECs were observed at different concentrations of heme treatment (**Figure 1A**), and a concentration of 40 µM was used in the subsequent experiments. Propidium iodide (PI) staining results indicated that more cells died after heme treatment and that hemopexin reversed this effect (**Figures 1B, C**). LDH release also increased after heme treatment, and hemopexin reversed this effect (**Figure 1D**). The levels of IL-1β and IL-18 in conditioned medium collected from HUVECs were also increased after heme treatment, and hemopexin reversed this effect (**Figure 1E**). Furthermore, we evaluated the expression of pyroptosis markers in HUVECs. Western blotting results showed that the pore-forming N-terminal fragment of gasdermin D (GSDMD-N) and Casp1 p20 increased after heme treatment and that hemopexin reversed this effect (**Figure 1F**). These results indicate that heme induces pyroptosis in endothelial cells and that hemopexin can rescue heme-induced pyroptosis of endothelial cells.

### 3.2 Hemopexin reverses heme-induced SIRS in mice

**Figure 2:**
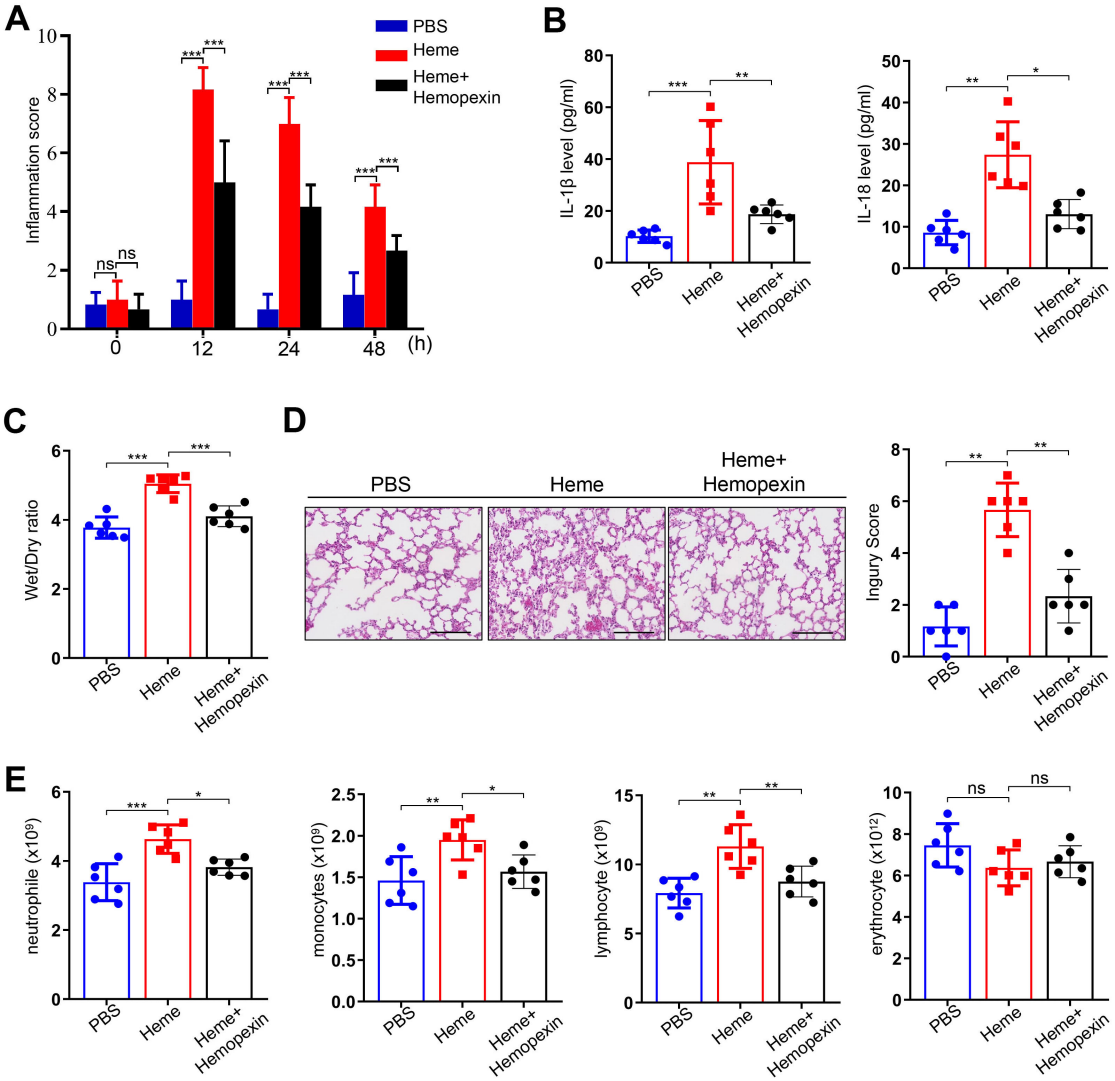
Hemopexin alleviates heme-induced SIRS in mice. **(A)** The inflammation score of mice was assayed at 0 h, 12 h, 24 h, and 48 h after treatment of heme and hemopexin (n=6, by one-way ANOVA). **(B)** The serum levels of IL-1β and IL-18 were detected after treatment of heme and hemopexin (n=6, by one-way ANOVA). **(C)** The wet-to-dry lung ratio was calculated after treatment of heme and hemopexin (n=6, by one-way ANOVA). **(D)** The pathological changes of lung tissues of mice were observed by HE staining after treatment of heme and hemopexin. Scale bar = 50 µm (n=6, by one-way ANOVA). **(E)** The neutrophile, monocyte, lymphocyte, and erythrocyte counts were detected after treatment of heme and hemopexin (n=6, by one-way ANOVA). The data are expressed as mean ± SEM. ns: no significance, *P < 0.05, **P < 0.01, ***P < 0.001.

To determine the effect of hemopexin on heme-induced SIRS, mice were divided into PBS, heme, and heme + hemopexin groups. The inflammation scores of the mice were analyzed at 0, 12, 24, and 48 h to evaluate SIRS after PBS, heme, and hemopexin administration. The results showed that the inflammation score significantly increased at 12 and 24 h in the heme group and decreased in the heme + hemopexin group (**Figure 2A**). Notably, the heme-induced increased levels of IL-1β and IL-18 in serum were significantly decreased in mice after treatment with hemopexin (**Figure 2B**). The wet-to-dry lung ratio also increased in the heme group and decreased significantly after hemopexin administration (**Figure 2C**). In addition, changes in lung tissue in different groups of mice were noted. The alveolar septum was significantly thickened, with numerous inflammatory cells infiltrating the tissue in the heme group, which was decreased significantly in the heme + hemopexin group (**Figure 2D**). Furthermore, the neutrophil, monocyte, and lymphocyte counts in the peripheral blood increased after heme treatment and decreased significantly after hemopexin administration (**Figure 2E**). Additionally, HE staining revealed that there were no significant alterations in heart, liver and spleen after heme treatment (**Supplementary Figure S1A**). Collectively, these results indicate that hemopexin reverses heme-induced SIRS in mice.

### 3.3 HMGB1 participates in heme-induced pyroptosis of endothelial cells

**Figure 3:**
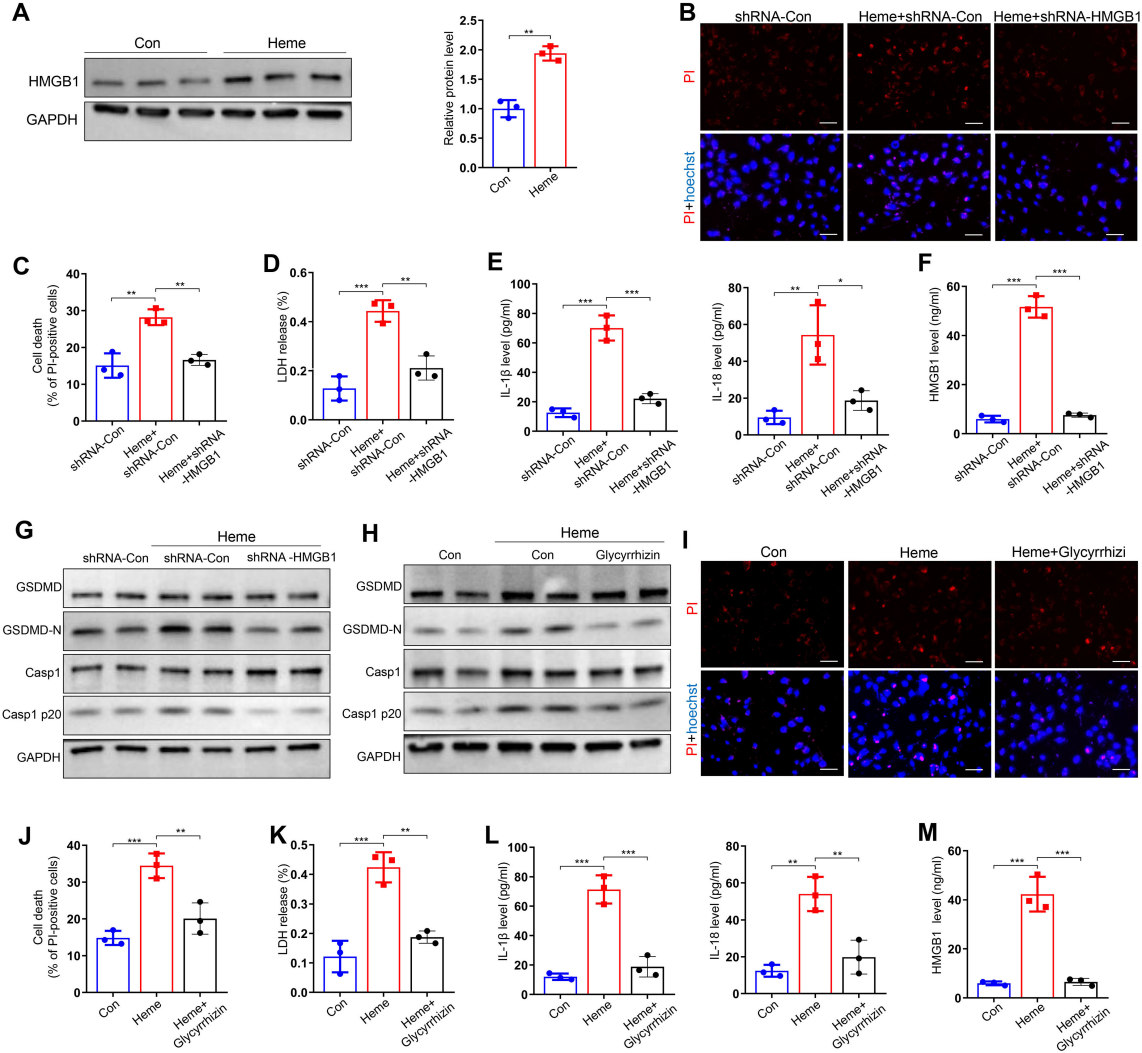
HMGB1 is involved in heme-induced pyroptosis of endothelial cells. **(A)** Protein level of HMGB1 in HUVECs was detected by Western blot assay after treatment of heme (n=3, by two-tailed Student’s *t*-test). **(B)** HMGB1 knockdown HUVECs were stained with PI and Hoechst for 20 min to observe cell death after heme treatment. Scale bar = 100 µm. **(C)** Cell death of HMGB1 knockdown HUVECs was assayed after heme treatment (n=3, by one-way ANOVA). **(D)** The level of LDH in supernatant of HMGB1 knockdown HUVECs was detected after heme treatment (n=3, by one-way ANOVA). **(E)** Levels of IL-1β and IL-18 in HMGB1 knockdown HUVECs supernatant were assayed by ELISA after heme treatment (n=3, by one-way ANOVA). **(F)** The level of HMGB1 in the supernatant of HMGB1 knockdown HUVECs was assayed by ELISA after heme treatment (n=3, by one-way ANOVA). **(G)** Protein levels of GSDMD, GSDMD-N, Casp1, and Casp1 p20 in HMGB1 knockdown HUVECs were detected by Western blot assay after heme treatment (n=3, by one-way ANOVA). **(H)** Protein levels of GSDMD, GSDMD-N, Casp1, and Casp1 p20 in HUVECs were detected by Western blot assay after treatment of heme and glycyrrhizin (n=3, by one-way ANOVA). **(I)** HUVECs were stained with PI and Hoechst for 20 min to observe cell death after treatment of heme and glycyrrhizin. Scale bar = 100 µm. **(J)** Cell death of HUVECs was assayed after treatment of heme and glycyrrhizin (n=3, by one-way ANOVA). **(K)** The level of LDH in supernatant of HUVECs was detected after treatment of heme and glycyrrhizin (n=3, by one-way ANOVA). **(L)** Levels of IL-1β and IL-18 in HUVECs supernatant were assayed by ELISA after treatment of heme and glycyrrhizin (n=3, by one-way ANOVA). **(M)** The level of HMGB1 in supernatant of HUVECs was assayed by ELISA after treatment of heme and glycyrrhizin (n=3, by one-way ANOVA).The data are expressed as mean ± SEM. ns: no significance, *P < 0.05, **P < 0.01, ***P < 0.001.

Previous studies have shown that HMGB1 plays a key role in pyroptosis (15), however, the mechanisms of HMGB1 in heme-induced pyroptosis of endothelial cells have not been reported. We evaluated the effect of HMGB1 on heme-induced pyroptosis. Western blotting results showed that HMGB1 was upregulated in HUVECs after heme treatment (**Figure 3A**). PI staining results revealed that HMGB1 knockdown suppressed the heme-induced death of HUVECs, and LDH release was also decreased in HMGB1 knockdown HUVECs after heme treatment (**Figures 3B–D**). HMGB1 downregulation also reversed the elevated levels of IL-1β, IL-18 and HMGB1 in conditioned medium from HUVECs after heme treatment (**Figures 3E, F**). Furthermore, HMGB1 knockdown downregulated GSDMD-N and Casp1 p20 expression in HUVECs, reversing the effects of heme (**Figure 3G**, **Supplementary Figure S2A**). Inhibition of HMGB1 using glycyrrhizin also downregulated the expression of GSDMD-N and Casp1 p20 in HUVECs (**Figure 3H**, **Supplementary Figure S2B**). Inhibition of HMGB1 also attenuated the effect of heme-induced death of HUVECs, and LDH release was decreased in HMGB1-inhibiting HUVECs after heme treatment by glycyrrhizin (**Figures 3I–K**). Notably, elevated IL-1β, IL-18 and HMGB1 levels in conditioned media from HUVECs after heme treatment were reversed after inhibiting HMGB1 by glycyrrhizin (**Figures 3L, M**). These results indicate that HMGB1 participates in heme-induced pyroptosis in endothelial cells. Furthermore, the protein level of HMGB1 in heme-treated HUVECs was detected after administration of hemopexin and HMGB1 inhibitors, including glycyrrhizin, Quercetin and Ethyl pyruvate. Hemopexin and glycyrrhizin exerted the most potent inhibitory effect on HMGB1 (**Supplementary Figure S3A**).

### 3.4 Heme regulates pyroptosis through the ROS/HMGB1/NLRP3 pathway

**Figure 4:**
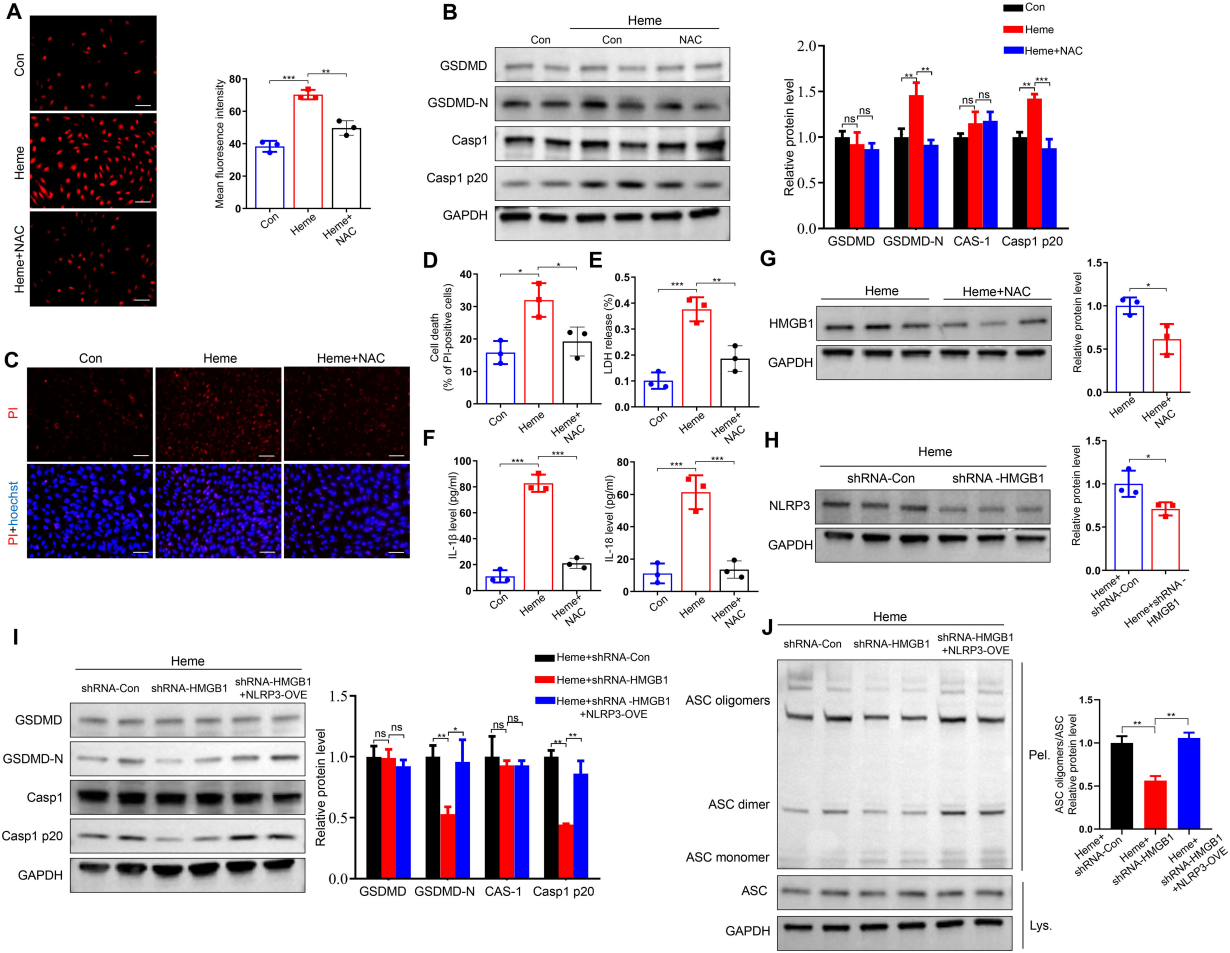
Heme regulates pyroptosis through ROS/HMGB1/NLRP3 pathway. **(A)** The ROS content in HUVECs was assayed after treatment of heme and NAC (n=3, by one-way ANOVA). Scale bar = 100 µm. **(B)** Protein levels of GSDMD, GSDMD-N, Casp1, and Casp1 p20 in HUVECs were assayed by Western blot assay after treatment of heme and NAC (n=3, by one-way ANOVA). **(C)** The HUVECs were stained with PI and Hoechst for 20 min to observe cell death after treatment of heme and NAC. Scale bar = 100 µm. **(D)** Cell death of HUVECs was assayed after treatment of heme and NAC (n=3, by one-way ANOVA). **(E)** The LDH level in supernatant of HUVECs was assayed after treatment of heme and NAC (n=3, by one-way ANOVA). **(F)** Levels of IL-1β and IL-18 in supernatant of HUVECs were assayed by ELISA after treatment of heme and NAC (n=3, by one-way ANOVA). **(G)** Protein level of HMGB1 in HUVECs with treatment of heme and NAC was detected by Western blot assay (n=3, by two-tailed Student’s *t*-test). **(H)** The protein level of NLRP3 in HMGB1 knockdown HUVECs was detected by Western blot assay after treatment of heme (n=3, by two-tailed Student’s *t*-test). **(I)** Protein levels of GSDMD, GSDMD-N, Casp1, and Casp1 p20 in HMGB1 knockdown and NLRP3 overexpression HUVECs were detected by Western blot assay after treatment of heme (n=3, by one-way ANOVA). **(J)** ASC oligomerization in cross-linked cytosolic pellets of HMGB1 knockdown and NLRP3 overexpression HUVECs was detected by Western blot assay after treatment of heme (n=3, by one-way ANOVA). The data are expressed as mean ± SEM. ns, no significance, *P < 0.05, **P < 0.01, ***P < 0.001.

Our previous study indicated that ROS generation was upregulated after heme treatment (16). We treated HUVECs with heme and ROS scavenger N-acetyl-l-cysteine (NAC). Heme-induced ROS upregulation is inhibited after NAC treatment (**Figure 4A**). NAC also inhibited heme-induced increases in GSDMD-N and Casp1 p20 levels in HUVECs (**Figure 4B**). The PI staining results indicated that NAC suppressed the heme-induced death of HUVECs, and LDH release was also decreased in heme-treated HUVECs after NAC administration (**Figures 4C–E**). In addition, the elevated levels of IL-1β and IL-18 in conditioned media from HUVECs after heme treatment were reduced after NAC administration (**Figure 4F**). A previous study revealed that the inhibition of ROS could suppress HMGB1 activation (17). Furthermore, we explored whether ROS regulates pyroptosis through HMGB1 in endothelial cells. Western blotting showed that NAC suppressed HMGB1 expression in heme-treated HUVECs (**Figure 4G**), indicating that heme-induced ROS could upregulate HMGB1 expression. Another previous study showed that HMGB1 could activate the downstream pyroptosis pathway in microglia (12). Therefore, we investigated whether HMGB1 could similarly activate this pathway in endothelial cells. HMGB1 knockdown significantly inhibited NLRP3 expression (**Figure 4H**). In addition, HMGB1 knockdown reduced GSDMD-N and Casp1 p20 expression in heme-treated HUVECs, whereas NLRP3 overexpression in HMGB1 knockdown HUVECs reversed this effect (**Figure 4I**). Furthermore, ASC speck formation triggered by heme was also inhibited in HMGB1 knockdown HUVECs, and NLRP3 overexpression in HMGB1 knockdown HUVECs reversed this effect (**Figure 4J**), indicating that NLRP3 is the downstream effector molecule of HMGB1. Collectively, these findings indicate that heme promotes pyroptosis through the ROS/HMGB1/NLRP3 pathway.

### 3.5 Glycyrrhizin and Hemopexin alleviate SIRS after RFA of hepatic hemangioma in mice

**Figure 5:**
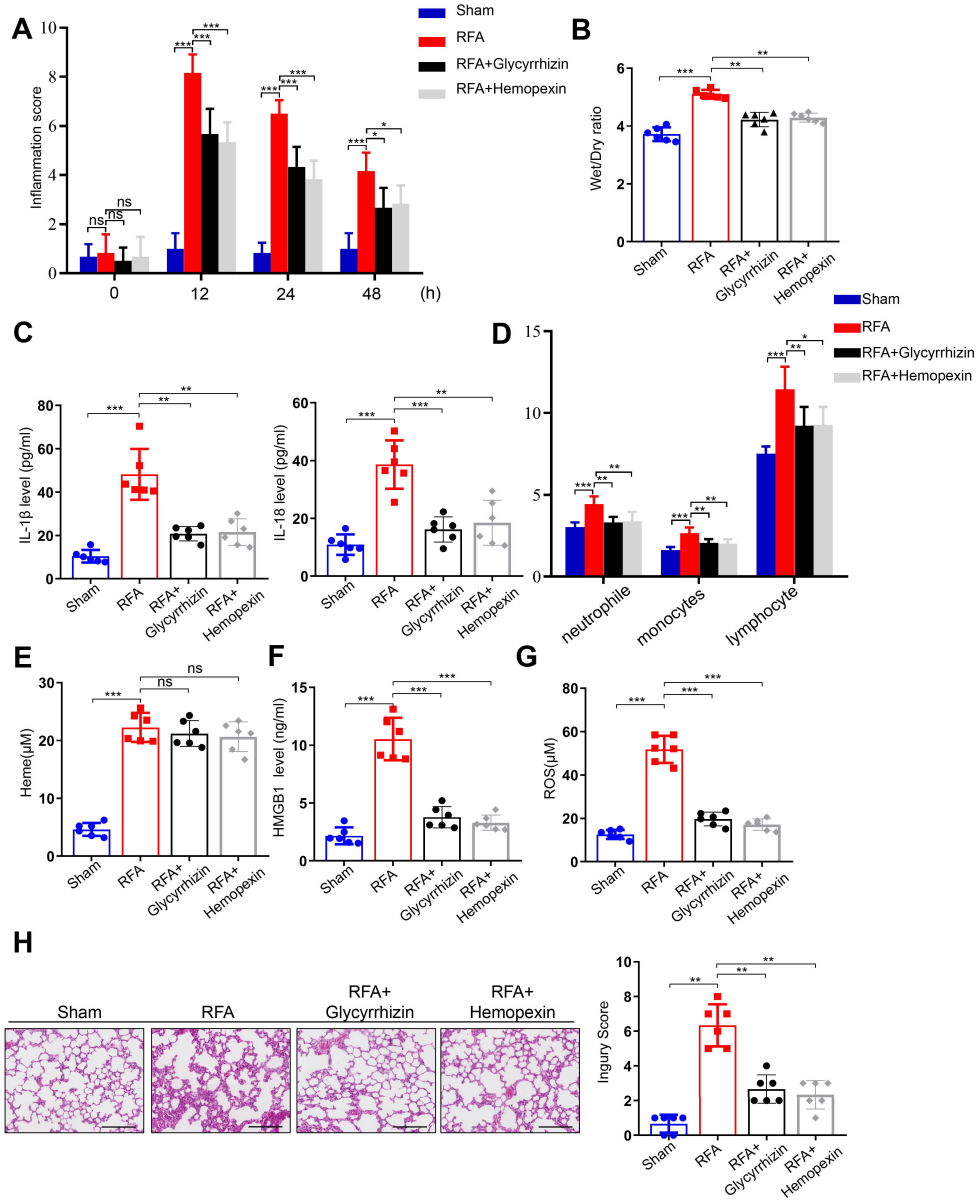
Glycyrrhizin and Hemopexin alleviate SIRS after RFA of hepatic hemangioma in mice. **(A)** The inflammation score of mice treated with glycyrrhizin or hemopexin was assayed at 0 h, 12 h, 24 h, and 48 h after RFA (n=6, by one-way ANOVA). **(B)** The wet-to-dry lung ratio of mice treated with glycyrrhizin or hemopexin was calculated after RFA (n=6, by one-way ANOVA). **(C)** The serum levels of IL-1β and IL-18 of mice treated with glycyrrhizin or hemopexin were detected by ELISA after RFA (n=6, by one-way ANOVA). **(D)** The neutrophile, monocyte, and lymphocyte counts of mice treated with glycyrrhizin or hemopexin were detected after RFA (n=6, by one-way ANOVA). **(E–G)** The serum levels of heme, HMGB1 and ROS of mice treated with glycyrrhizin or hemopexin were detected by ELISA after RFA (n=6, by one-way ANOVA). **(H)** The pathological changes of lung tissues of mice treated with glycyrrhizin or hemopexin were observed by HE staining after RFA (n=6, by one-way ANOVA). Scale bar = 50 µm. The data are expressed as mean ± SEM. ns: no significance, *P < 0.05, **P < 0.01, ***P < 0.001.

In this study, we developed an orthotopic liver hemangioma model. To clarify the effects of the solvent vehicle, RFA + vehicle group was included alongside the RFA group. The results showed that there was no significant difference between the two groups (**Supplementary Figures S4A-D**). Then, mice with orthotopic liver hemangioma were divided into four groups: sham, RFA, RFA + glycyrrhizin, and RFA + hemopexin. Inflammation scores were assessed, which significantly increased in the RFA group. However, glycyrrhizin or hemopexin administration reversed this effect in mice after RFA of hemangiomas (**Figure 5A**). The wet-to-dry lung ratio increased in the RFA group but was reversed by glycyrrhizin or hemopexin administration (**Figure 5B**). In addition, we measured the serum levels of IL-1β and IL-18 in mice. The levels of IL-1β and IL-18 increased in the RFA group but decreased in the RFA + glycyrrhizin and RFA + hemopexin groups (**Figure 5C**). Furthermore, we assessed neutrophil, monocyte, and lymphocyte counts in the peripheral blood of the mice. Notably, neutrophil, monocyte, and lymphocyte counts increased significantly after RFA and decreased in the RFA + glycyrrhizin and RFA + hemopexin groups (**Figure 5D**). We also assessed serum heme, HMGB1 and ROS of mice in different groups. The level of heme increased after RFA, however, heme didn’t decrease in the RFA + glycyrrhizin and RFA + hemopexin group, indicating that glycyrrhizin and hemopexin could not inhibit the production of heme after RFA (**Figure 5E**). The levels of HMGB1 and ROS in serum of mice also increased in the RFA group, but decreased in the RFA + glycyrrhizin and RFA + hemopexin groups (**Figures 5F, G**). Moreover, the alveolar septum showed significant thickening with numerous infiltrated inflammatory cells in the RFA group, which was significantly decreased in the RFA + glycyrrhizin and RFA + hemopexin groups (**Figure 5H**). Additionally, to confirm that endothelial cells are the primary cells inducing pyroptosis in heme treated mice, immunofluorescence of CD31 and NLRP3 was performed. Co-localization of NLRP3 and CD31 in vascular endothelial cells of mouse lung tissue was observed after heme treatment, while no notable NLRP3 expression was observed in other cells, indicating that endothelial cells are the primary cell type contributing to pyroptosis in mice of heme-induced SIRS (**Supplementary Figure S5A**). In summary, these findings indicate that targeting heme or HMGB1 could alleviate SIRS after RFA of hepatic hemangiomas in mice.

## 4 Discussions

RFA is increasingly considered a safe and effective alternative treatment to traditional surgical resection for hepatic hemangiomas. However, SIRS often occurs in patients after RFA treatment for hepatic hemangiomas, particularly with large hemangiomas (2). Our previous study identified heme as a key factor contributing to SIRS after RFA for hepatic hemangiomas (6). The present findings demonstrated that heme-induced upregulation of HMGB1 results in endothelial cell pyroptosis and plays a critical role in this process. These findings reveal a potential novel strategy for preventing and treating SIRS after RFA in patients with hepatic hemangiomas.

Heme is a DAMP that interacts with receptors such as Toll-like receptors (TLRs), leading to autoimmune reactions or immune tolerance, and plays a significant role in inflammation and immune responses (18–21). Free heme can cause various harmful effects, including the promotion of ROS and inflammatory mediators such as tumor necrosis factorα (TNF-α), as well as the increased expression of endothelial cell adhesion molecules (22–26). Our previous study revealed that heme levels increase after RFA of hepatic hemangiomas and that circulating heme contributes to the induction of SIRS (16). Hemopexin, a heme scavenger, could prevent heme induced injury by binding to heme and allows the transport of heme in the body in a nontoxic form and promotes heme detoxification in the liver. In the present study, hemopexin inhibited heme-induced pyroptosis *in vitro* and heme-induced SIRS *in vivo.*

HMGB1, a classic signaling molecule, plays diverse biological roles in pathological and pathophysiological processes, including immune responses, inflammation, cell differentiation, angiogenesis, and cell death (27). HMGB1 exhibits potent inflammatory effects and functions as a DAMP when released from cells. In addition, it can induce significant production of inflammatory cytokines in immune cells through various signaling pathways, including cell surface pattern recognition receptors such as Toll-like receptors-2 (TLR-2), TLR-4, and receptor for advanced glycation end products (RAGE), thereby contributing to SIRS. In a previous study, HMGB1 was shown to initiate endocytosis and trigger a series of molecular events, including the release of cathepsin B from ruptured lysosomes. This release leads to the activation of caspase-1, ultimately resulting in macrophage pyroptosis through RAGE-dependent signaling. Notably, HMGB1-induced pyroptosis has been observed *in vivo* during endotoxemia (28). It is still unclear whether heme regulates HMGB1 expression during pyroptosis in endothelial cells. Wagener. et al. reported that heme increases endothelial permeability and decreases macrophage-dependent bacterial clearance through an HMGB1-dependent mechanism (29). Lin. et al. reported that administering hemopexin to prevent heme elevation is beneficial in HMGB1-induced sterile and infectious inflammation (30). In our study, we found that heme-induced HMGB1 upregulation resulted in pyroptosis in endothelial cells.

Glycyrrhizin, also known as glycyrrhizic acid, is a naturally occurring compound found in the licorice plant *Glycyrrhiza glabra*, which can directly bind to and inhibit HMGB1 (31). Studies investigating the therapeutic potential of glycyrrhizin have reported promising results in the treatment of inflammatory diseases, including intracerebral hemorrhage, ischemia–reperfusion injury, sepsis, and cancer. One previous study showed that glycyrrhizin significantly reduced serum levels of HMGB1 *in vivo*. Furthermore, glycyrrhizin inhibits HMGB1-mediated chemotaxis by blocking the HMGB1–CXCL2 heterocomplex after muscle injury (32). In our study, we observed that glycyrrhizin alleviated SIRS after RFA in hepatic hemangiomas.

Our study has several limitations. The experiments were conducted only in mice, whose pathophysiology may differ from that of humans. The use of diverse animal models and the assessment of various doses of glycyrrhizin are critical for determining its optimal therapeutic regimen. Additionally, glycyrrhizin may have off-target effects. Therefore, further experiments and improved delivery systems for glycyrrhizin are necessary.

## 5 Conclusion

This study revealed that heme regulates HMGB1 expression and induces SIRS after RFA of hepatic hemangiomas through ROS. In addition, the heme-binding protein hemopexin and the HMGB1 inhibitor glycyrrhizin alleviated heme-induced SIRS in mice. Moreover, increased ROS production resulted in the upregulation of HMGB1, which in turn activated the NLRP3 inflammasome. Therefore, heme-mediated endothelial cell pyroptosis induces SIRS through the ROS/HMGB1/NLRP3 pathway after RFA of hepatic hemangiomas. Collectively, these findings indicate that hemopexin and glycyrrhizin may be the potential strategies for further study for SIRS following RFA of hepatic hemangioma.

## Data Availability

The raw data supporting the conclusions of this article will be made available by the authors, without undue reservation.
