# Supplementary material for: Targeting HMGB1 in endothelial cells reverses heme-induced SIRS after radiofrequency ablation of hepatic hemangioma
Source: Front Immunol. 2025 Nov 6;16:1680433. doi: 10.3389/fimmu.2025.1680433 (PMC12631351; doi:10.3389/fimmu.2025.1680433)
Supplement: Supplementary file 1 [file DataSheet1.docx]

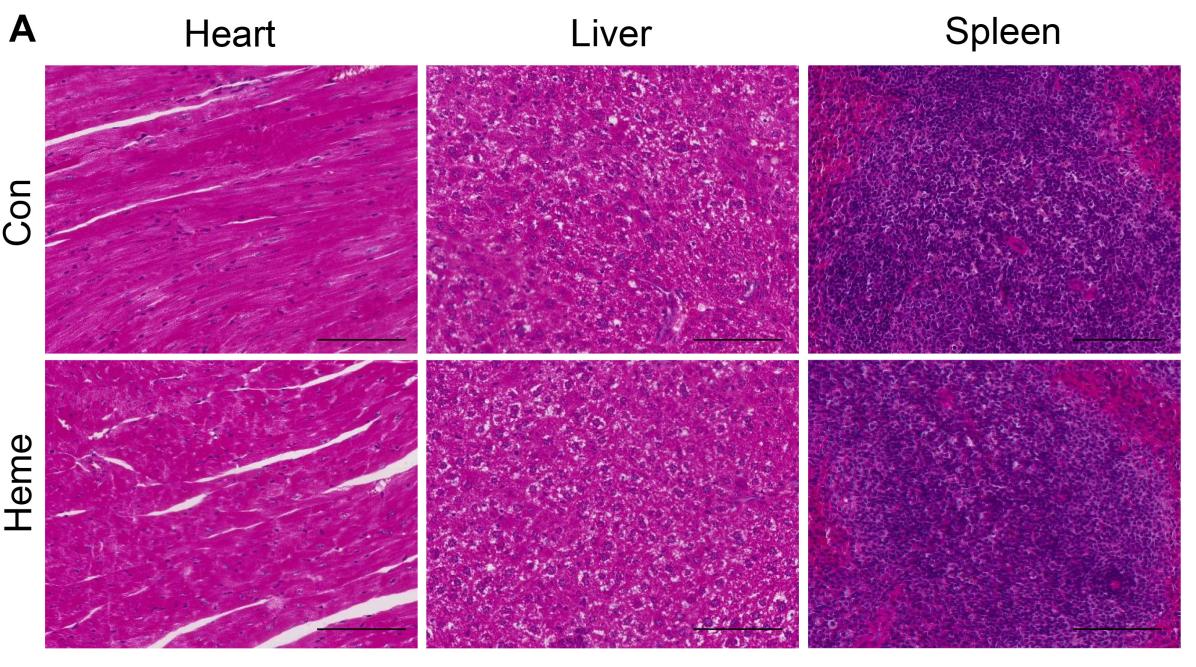
Supplementary Material

**Supplementary Figure 1 (A)** HE staining of heart, liver, and spleen of mice after heme treatment. Scale bar = 50 µm.


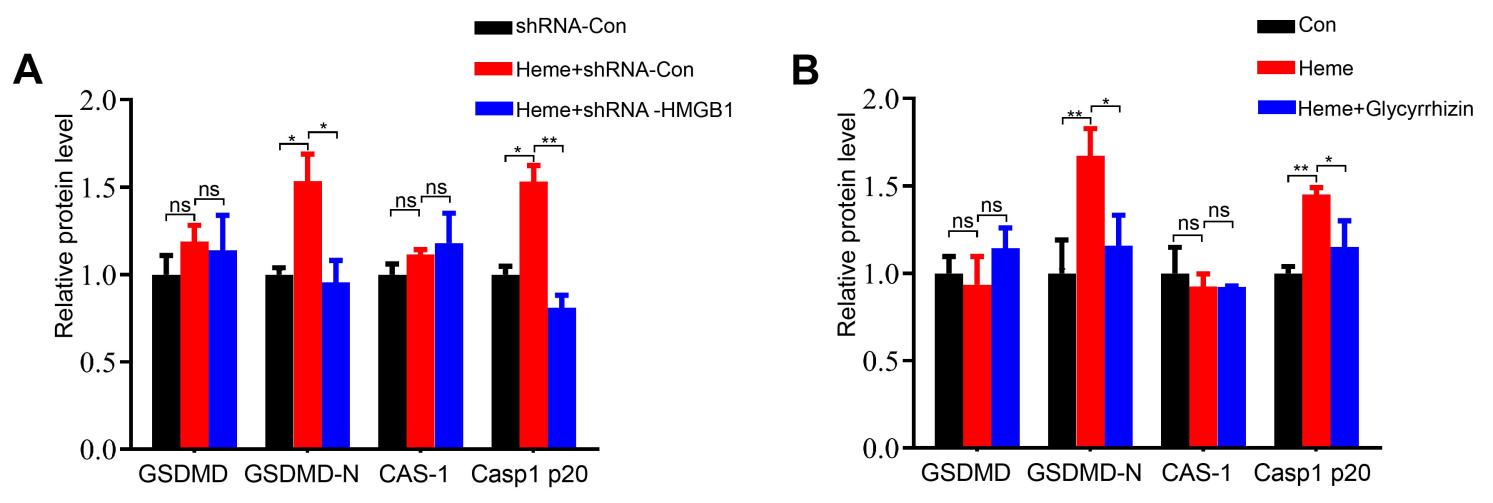


**Supplementary Figure 2 (A)** Statistical analysis of protein levels of GSDMD, GSDMD-N, Casp1, and Casp1 p20 in HMGB1 knockdown HUVECs detected by Western blot assay after heme treatment (n=3, by one-way ANOVA). **(B)** Statistical analysis of protein levels of GSDMD, GSDMD-N, Casp1, and Casp1 p20 in HUVECs detected by Western blot assay after treatment of heme and glycyrrhizin (n=3, by one-way ANOVA). The data are expressed as mean ± SEM. ns: no significance, *P<0.05, **P<0.01, ***P<0.001.


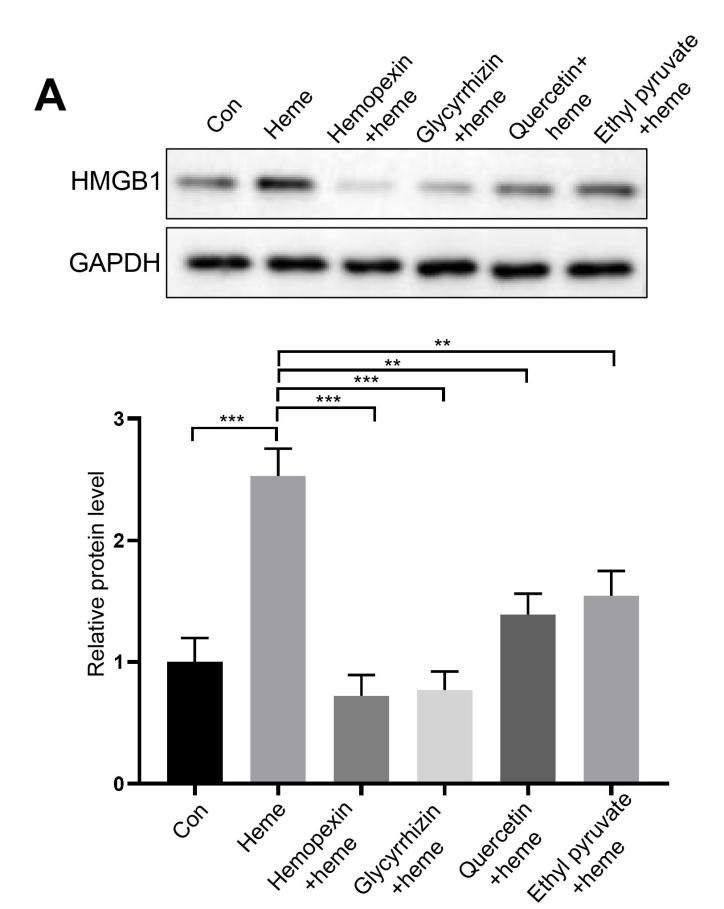


**Supplementary Figure 3 (A)** Protein level of HMGB1 in heme-treated HUVECs were detected by Western blot assay after adminstration of hemopexin, glycyrrhizin, quercetin and ethyl pyruvate. (n=3, by one-way ANOVA). The data are expressed as mean ± SEM. **P<0.01, ***P<0.001.


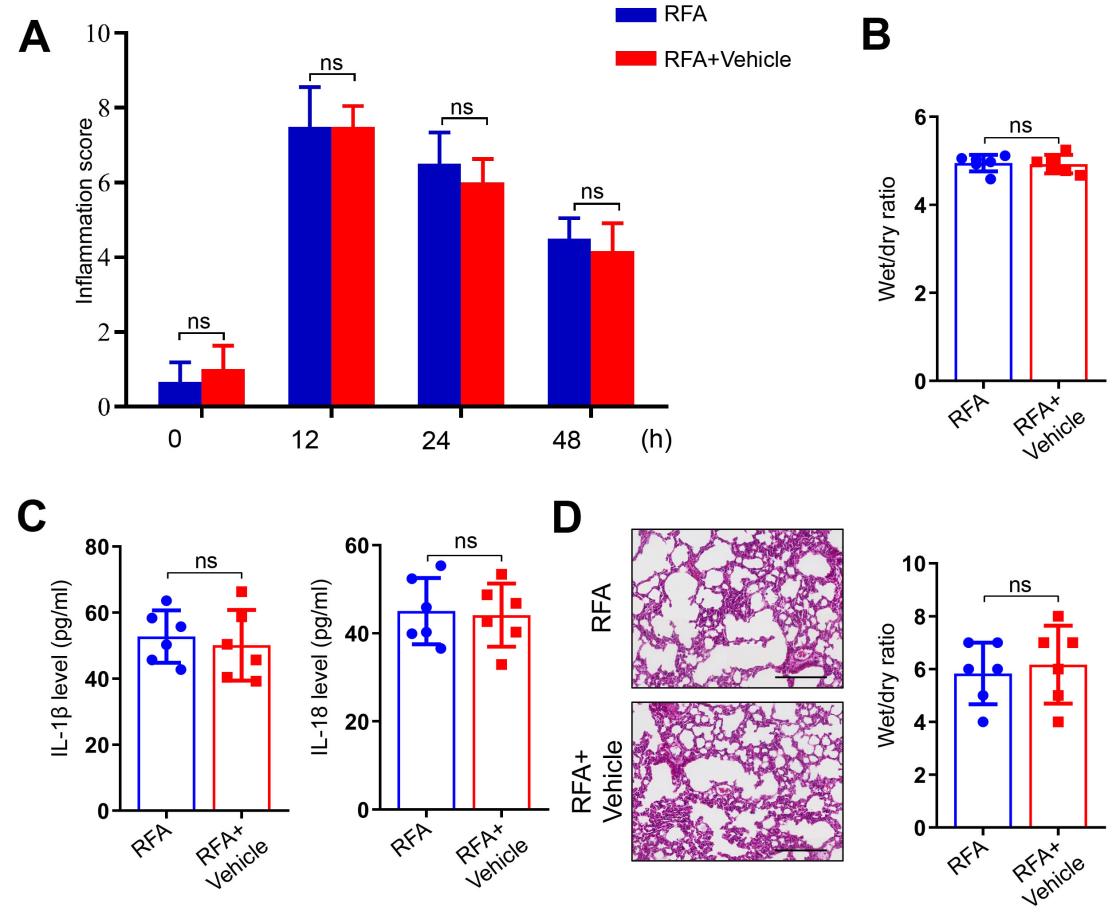


**Supplementary Figure 4 (A)** The inflammation score of mice in RFA group and RFA+vehicle group was assayed at 0 h, 12 h, 24 h, and 48 h (n=6, by one-way ANOVA). **(B)** The wet-to-dry lung ratio of mice in RFA group and RFA+vehicle group was assayed (n=6, by one-way ANOVA). **(C)** The serum levels of IL-1β and IL-18 of mice in RFA group and RFA+vehicle group were assayed by ELSA (n=6, by one-way ANOVA). **(D)** The pathological changes of lung tissues of mice in RFA group and RFA+vehicle group were observed by HE staining (n=6, by one-way ANOVA). Scale bar = 50 µm. The data are expressed as mean ± SEM. ns: no significance.


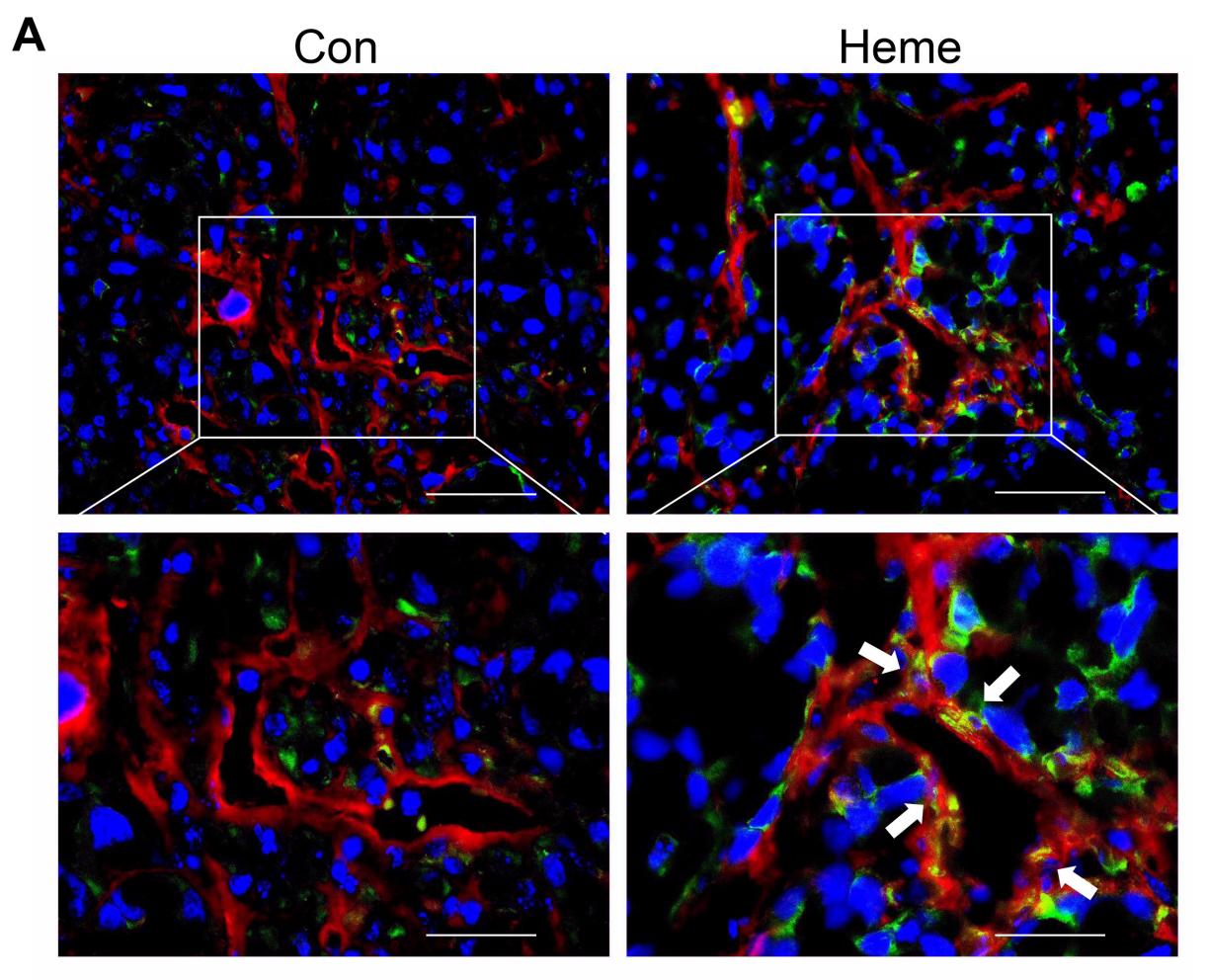


**Supplementary Figure 5 (A)** Immunofluorescence staining for NLRP3 (green), CD31 (red) and DAPI (blue) in heme treated HUVECs, the arrow shows the colocalization of NLRP3 and CD31. Scale bar = 20 µm.
